# A robust model of sensory tuning using dendritic non-linearities

**DOI:** 10.1186/1471-2202-16-S1-P223

**Published:** 2015-12-18

**Authors:** Romain D Cazé, Sarah Jarvis, Simon R Schultz

**Affiliations:** 1 Centre for Neurotechnology & Department of Bioengineering, Imperial College London, London, UK

## 

Dendrites, like neurons, can preferentially activate for certain stimuli, but recent experimental evidence suggests that dendritic tuning can differ from the neuronal tuning. For instance, in a L2/3 pyramidal neuron in the mouse visual cortex, dendritic calcium signals display a wide range of tuning profiles, some of which differ from the tuning of the neuronal output [1]. This puzzling observation was unanticipated by the standard Hubel and Wiesel model explaining the origin of visual tuning [2]. The standard model can survive this observation, but only with the addition of superfluous synapses. We propose here an alternative model where synapses responsible for neuronal tuning are dispersed over dendrites. This alternative model builds on previously published results [3]. It possesses non-linear dendritic compartments, and in each compartment the result of multiple excitatory inputs can be smaller than their arithmetic sum. These non-linear and independent sites of synaptic integration create neuronal tuning: groups of correlated presynaptic inputs encode the stimulus identity, and only the group that encodes the preferred stimulus targets different dendrites and leads to a response. Groups coding for non-preferred stimuli instead target the same dendrite, and explain the wide range of dendritic tunings observed experimentally. Moreover, we demonstrate that this implementation of neuronal tuning is robust to the loss of dendrites. Thus, our alternative model not only reproduces the experimental observations, but is also robust to dendritic reorganization. To confirm this result *in silico *we use a multi-compartmental model with a realistic L2/3 morphology [1] (see Figure 1). Our work implies that non-linear integration in dendrites can play a pivotal role in neuronal tuning even if dendritic and neuronal tuning differ. This implementation of neuronal tuning is also robust to dendritic loss, a property important to keep a stable sensory representation. Importantly, our theoretical framework predicts that a neuron is tuned to the input generating the most widespread synaptic activity on its dendrites and that neuronal tuning can resist the loss of a significant number of dendrites.

**Figure 1:**
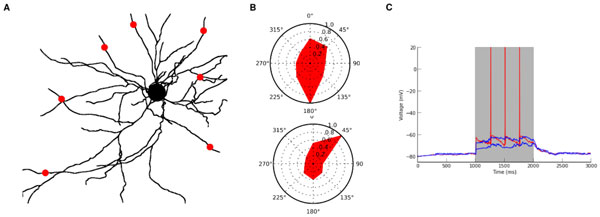
**Representation of the biophysical model used in the simulation**. Red dots indicate the 7 input sites. The black dot indicates the soma. **1B**. Polar plot showing the tuning of the membrane voltage within two dendritic compartments of our model. **1C**. Somatic voltage for three different stimuli (0, 45, 90 degrees). Red indicates the preferred stimulus, and blue traces non-preferred stimuli.

## References

[B1] Jia H Rochefort NL Chen X Konnerth A Dendritic organization of sensory input to cortical neurons in vivo Nature 2010 464 1307 1312 20428163 10.1038/nature08947

[B2] Hubel D Wiesel T Receptive fields, binocular interaction and functional architecture in the cat's visual cortex J Physiol 1961 160 1 106 154 14449617 10.1113/jphysiol.1962.sp006837 PMC1359523

[B3] Cazé RD Humphries M Gutkin B Passive Dendrites Enable Single Neurons to Compute Linearly Non-separable Functions PLoS Comput. Biol 2013 9 e1002867 23468600 10.1371/journal.pcbi.1002867 PMC3585427

